# Zoledronic acid inhibits TSC2-null cell tumor growth via RhoA/YAP signaling pathway in mouse models of lymphangioleiomyomatosis

**DOI:** 10.1186/s12935-020-1131-4

**Published:** 2020-02-10

**Authors:** Dandan Zhao, Jing Wu, Yinjuan Zhao, Wei Shao, Qi Cheng, Xiaoyan Shao, Xianwen Yuan, Juan Ye, Jianpu Gao, Meiling Jin, Chaojun Li, Xiaolin Chen, Yue Zhao, Bin Xue

**Affiliations:** 1 Core Laboratory, Sir Run Run Hospital, Nanjing Medical University, Nanjing, 211166 Jiangsu China; 2 State Key Laboratory of Pharmaceutical Biotechnology, Jiangsu Key Laboratory of Molecular Medicine and School of Medicine, Nanjing University, Nanjing, 210093 Jiangsu China; 3 Collaborative Innovation Center of Sustainable Forestry in Southern China, College of Forestry, Nanjing Forestry University, Nanjing, 210037 Jiangsu China; 4 Department of Hepatobiliary Surgery, The Affiliated Drum Tower Hospital of Nanjing University Medical School, Nanjing, 210008 Jiangsu China; 5 Affiliated Hospital of Integrated Traditional Chinese and Western Medicine, Nanjing University of Chinese Medicine, Nanjing, 210028 Jiangsu China; 6 Pulmonary Department, Zhongshan Hospital, Fudan University, Shanghai, 200032 China; 7 Jiangsu Key Laboratory of Molecular Medicine and School of Medicine, Nanjing University, Nanjing, 210093 Jiangsu China; 8 State Key Laboratory of Natural Medicines, China Pharmaceutical University, Nanjing, 210009 Jiangsu China; 9 Respiratory Department, Sir Run Run Hospital, Nanjing Medical University, Nanjing, 211166 Jiangsu China

**Keywords:** Lymphangioleiomyomatosis, MTOR, Zoledronic acid (ZA), YAP, Autophagy

## Abstract

**Background:**

This study is to investigate the effects of zoledronic acid (ZA) on TSC2-null cell proliferation and on the tumor progression and recurrence in mouse models of lymphangioleiomyomatosis (LAM).

**Methods:**

Subcutaneous mouse models and LAM mouse models were established. Immunohistochemistry and immunofluorescence were performed to detect the protein expression levels. TUNEL assay was conducted to detect cell apoptosis. Immunoprecipitation was carried out to determine the interaction between proteins.

**Results:**

ZA prevented the growth of TSC2-null cells both in culture and in LAM mouse models. Compared with rapamycin, ZA more effectively promoted the apoptosis of TSC2-null cells. Moreover, combined with the rapamycin, ZA effectively suppressed the tumor recurrence after drug withdrawal and ZA inhibited the activity of GTPase RhoA by decreasing protein geranylgeranylation, resulting in changes of Yap nucleus translocation.

**Conclusion:**

ZA promotes cell apoptosis in TSC2-null cells through the RhoA/YAP signaling pathway. ZA may be used for the clinical treatment of LAM.

## Background

Lymphangioleiomyomatosis (LAM) is a destructive cystic lung disease caused by LAM cells, which invade all the lung structures, including the lymphatics, airway wall, blood vessels, and interstitial spaces, therefore limiting the delivery of oxygen to the body. Moreover, LAM is a rare, progressive mesenchymal neoplasm mainly affecting childbearing age women [1–4]. The LAM cases are sometime difficult to diagnose because the disease symptoms are similar to other lung diseases (such as asthma, emphysema, and bronchitis). The high-resolution chest CT scan and VEGF-D blood test could provide accurate diagnosis of LAM [5, 6]. Each year, 3.3–7.4 million women have been diagnosed, and 0.23–0.31 million new cases have been confirmed [7]. Up to now, there is still no therapy that could cure LAM, but only the lung transplantation can save the life of LAM patients. In 2000–2006, some studies have found that somatic mutation of tuberous sclerosis complex 2 (TSC2) gene in LAM patients would cause abnormal proliferation of LAM cells [8, 9]. However, how the TSC2 mutation induces LAM and where these abnormally proliferated LAM cells come from have not yet fully elucidated.

LAM can be sporadic or co-exist with TSC, and all the cases have been found to be associated with function loss of *TSC2* gene. The TSC2 protein can form a complex with GTPase Rheb to block the activity of mTORC1, thus inhibiting the mTOR pathway [10]. Hence, the loss function mutation of *TSC2* gene in LAM patients causes the advanced activation of the mTORC1/mTORC2 pathways. The mTORC1 can phosphorylate several down-stream substrates, including the pS6 (Ser235/236) and 4EBP-1. Phosphorylation of the ribosomal protein S6 is increased in the LAM lung tissues, compared with the normal lung tissues [11]. The mTORC2 has been found to be insensitive to rapamycin, which could phosphorylate AKT at the Ser473 site and negatively regulate the AKT/PI3K pathway [12]. Rapamycin and rapalogs are the inhibitors of mTORC1, enhancing the rapid clinical transformation and benefiting many women with LAM [13, 14]. However, some clinical studies have shown that the lung function would become deteriorated again and relapse would occur after the cessation of rapamycin for 24 months [15, 16]. Moreover, Morris et al. [17] have reported that although mTORC1 is sensitive to rapamycin, mTORC2 is only sensitive to prolonged rapamycin treatment, and there might be drug tolerance. Therefore, multi-drug combination represents an effective method for the treatment of LAM. The mevalonate pathway is an essential metabolic pathway, in which the acetyl-CoA is used to produce sterols and isoprenoids (GGPP and FPP), involved in tumor growth and progression [18]. Bisphosphonate and statins are inhibitors of the mevalonate pathway, with however different target sites [19]. Statins are widely used HMG-CoA reductase inhibitors which affect many cross-talk pathways. Zoledronic acid (ZA) targets on two key enzymes in the mevalonate pathway, i.e., the FPPS and GGPPS, which are essential for the GTPase activity [20]. Several studies have reported that the combination of sivastatin and rapamycin represent effective antitumor intervention in the TSC2-null cells and the LAM mouse model. Moreover, some preliminary clinical researches have also shown that the rapamycin and stains treatments could improve the situations of some TCS and LAM patients [21]. However, long-term administration of statins would induce undesirable side effects, including abnormal lung function [22]. Furthermore, ZA has been found to exert long-term efficacy and safety in the treatment of skeletal metastases with non-small cell lung carcinoma and other solid tumors, without toxicity for patients, such as breast cancer and prostate cancer [23, 24].

In this study, the effects of ZA on TSC2-null cell proliferation and on the tumor progression and recurrence in LAM mouse models, were investigated. Moreover, the underlying mechanisms of ZA inducing effects in TSC2-null cells, and the involvement of the RhoA/YAP pathway were also analyzed. Our findings might provide evidence for the application of ZA in the clinical treatment of LAM.

## Methods

### Cell growth and viability assessment

TSC2-null MEF cells (Tsc2^−/−^p53^−/−^) were kindly gift from John Blenis (Weill Cornell Medical College, New York, NY, USA). These cells were cultured in the DMEM medium, supplemented with 10% FBS and 1% antibiotics. The viability of TSC2-null cells was detected by the MTT method. Briefly, 5 × 10^3^ cells were seeded onto 96-well plates and treated with indicated concentrations of ZA. After 24 h, 50 μl 1 × MTT solution (diluted by dilution buffer, KeyGENBioTECH) was added and incubated for 4 h. Then, 150 μl DMSO was added. The absorbance at 550 nm was measured by the Multiscan Spectrum.

### Animals and treatments

The 6–8-week-old STOCK-*Foxn1nu/*Nju female nude athymic mice were brought from the Model Animal Research Center of Nanjing University (Nanjing, Jiangsu, China). All animal procedures were carried out according to the guideline of the Animal Care and Use Committee of the Model Animal Research Center of Nanjing University. Mice were housed in a 12-h light/dark cycle, with free access to food and water. Animals did not suffer unnecessarily at any stage of an experiment.

### Subcutaneous mouse model establishment

For the subcutaneous model establishment, female nude mice were subcutaneously injected with 5 × 10^6^ TSC2-null cells in right flank. When tumors grew to 3 mm in diameter, the mice were injected with ZA (1 mg/kg/3 day; dissolved in 30% PEG400) through the tail vein, for 30 day. For the control group, mice were injected with placebo dissolved in 30% PEG400 through tail vein. Tumor volumes were calculated according to the following formulation: Tumor volume = Length × Width^2^ × 0.5. Animal weight were monitored and recorded.

### LAM mouse model establishment

For the establishment of LAM mouse models, female nude mice were subcutaneously injected with 5 × 10^6^ TSC2-null cells in both flanks, for 30–40 day. When the tumor volume reached 1 cm in diameter, mice were sacrificed. The tumors were removed and enzymatically digested with Collagenase Type IV and cultured with DMEM medium, supplemented with 10% FBS and 1% antibiotics. After 2 day, TSC2-null cells were harvested from the dishes and sifted through mesh, and 1 × 10^6^ cells were injected into the tail vein of female nude mice [25].

For both models, mice were injected with rapamycin (1 mg/kg/3 day), ZA (200 μg/kg/2 day and 1 mg/kg/3 day, respectively), or combination of ZA (1 mg/kg/3 day) and rapamycin (1 mg/kg/3 day). Rapamycin were injected intraperitoneally and ZA were injected through the tail vein. Animal weights were monitored and recorded. To determine whether the withdrawal of ZA would induce the tumor recurrence, the rapamycin treatment alone and the combination of rapamycin and ZA for 30 day was terminated. After 1 m, the animals were sacrificed to check the tumor recurrence.

### Immunohistochemistry detection

Mouse tissues were fixed in 4% paraformaldehyde, embedded in paraffin, and cut into 5-μm sections. Tissue sections were deparaffinized and hydrated before being incubated in 10 mmol/l citrate buffer (pH = 6) for 10 min in a microwave oven. Sections were blocked with goat serum for 1 h, and then incubated with indicated primary antibodies (Additional file 1: Table S1) at 4 °C overnight. Next, the sections were incubated with biotinylated secondary antibodies, and then immunoreactions were revealed by the DAB chromogen system.

### Immunofluorescence

Cell slides were fixed with 4% paraformaldehyde, washed with PBS, and then permeabilized with 0.1% Triton-100 for 15 min. Next, slides were blocked in goat serum for 1 h, and then incubated with the indicated primary antibodies (Additional file 1: Table S1) at 4 °C overnight. Alex Fluor 594- and Alex Flour 488-conjugate secondary antibodies were used. After stained with DAPI, immunofluorescence was detected.

### TUNEL assay

Apoptotic cells were detected by using DeadEnd™ Fluorometric TUNEL System (Promega, Madison, WI, USA), according to the manufacturer’s instructions. Immunofluorescence results was analyzed using the Image-Pro Expression system and the ImageJ software.

### Immunoblotting analysis

Cells were lysed in cell lysis buffer (RIPA) with complete protease inhibitor cocktail (Roche, Pleasanton, CA, USA) and centrifuged at 120,000*g* at 4 °C for 15 min. The cytosolic fraction in the supernatant was collected. Protein extract was separated by SDS-PAGE, and then electronically transferred onto the PVDF membrane (Roche). After blocking, the membrane was incubated with indicated primary antibodies (Additional file 1: Table S1) at 4 °C for overnight. After washing, the membrane was incubated with secondary antibody. Subsequently, immunoblotting was performed using the chemiluminescence method (Millipore, Bedford, MA, USA), according to manufacturer’s instructions.

### Immunoprecipitation

Membrane and cytoplasmic protein extracts were prepared with the Triton X-114 partition method [26]. These membrane and cytoplasmic proteins were immunoprecipitated using the RhoA protein antibody (Santa Cruz, Santa Cruz, CA, USA), followed by the Western blot analysis.

### Statistical analysis

All data were presented as mean ± SEM. Comparison between groups was performed using the Student’s *t* test. *P* < 0.05 was considered statistically significant.

## Results

### ZA exerts antitumor effects in TSC2-null cell subcutaneous tumor model and LAM mouse model

**Fig. 1:**
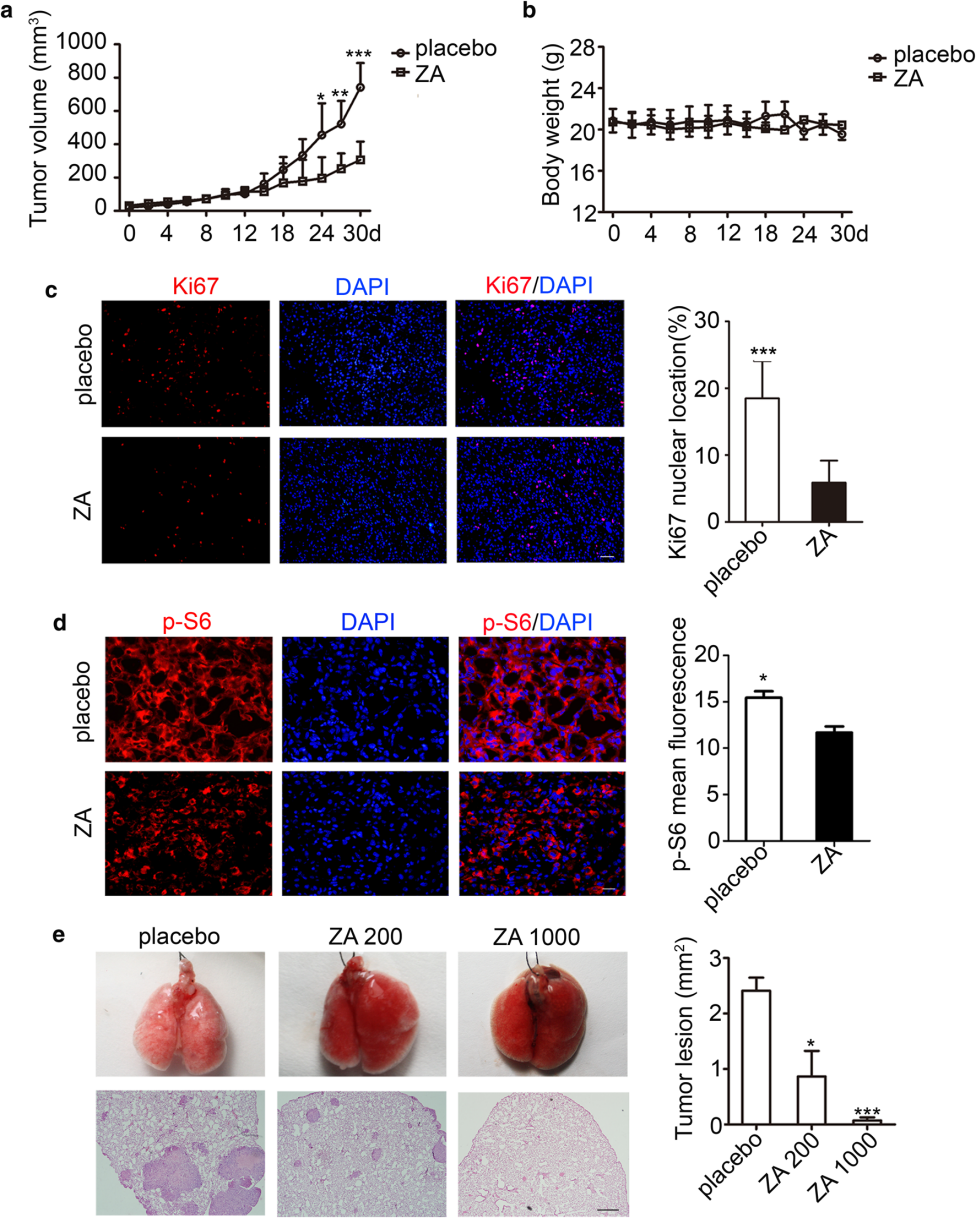
ZA exerts antitumor activity in TSC2-null cell subcutaneous tumor model and LAM mouse model. **a** Tumor growth in STOCK-Foxn1nu/Nju female athymic nude mice with TSC2-null cells given by 1 mg/kg/3 day zoledronic acid (ZA) through the tail vein (n = 6). **b** Body weights of nude mice with TSC2-null cell tumors were subjected to 30-d treatment with placebo and ZA, respectively (n = 6). **c** Immunofluorescence of Ki67 in the subcutaneous mice model. Scar bar, 100 μm (n = 6). **d** Tumor lesion sections stained for p-S6 (ser 234/235) (n = 6). Scar bar, 50 μm. **e** H&E staining of lung lesions of placebo- and ZA-injected mice after tail vein injection of TSC2-null cells for 30 day (n = 6). Scar bar, 500 μm. The experiment was performed for three times. All data were presented as mean ± SEM. Compared with the placebo, ** *P* < 0.01, *** *P *< 0.001

The antitumor potential of ZA on subcutaneous tumor model was first examined. The nude mice were subcutaneously injected with TSC2-null cells, and then treated with ZA (1 mg/kg/3 day; through tail vein injection). The doses of ZA were determined according to the clinical application for patients with bone disorders. To determine the effects of ZA on TSC2-null tumor growth, the tumor size and mouse weights were monitored and recorded three times a week. Our results showed that, after the ZA treatment, no significant changes were observed in the general behavior and mouse weight between the placebo and ZA groups. However, the tumors grew more quickly in the placebo group compared with the ZA treatment group, with significant difference for the time point of 3 weeks (Fig. 1a, b). Because the tumor volumes in the ZA group were decreased, the Ki67 staining was performed for the tumor tissues at 30 day after ZA treatment. Our results showed that the ZA treatment efficiently inhibited the TSC2-null cell proliferation (Fig. 1c).

To further confirm whether ZA influenced the mTORC1 activity in TSC2-null cells just like rapamycin, the phosphorylation of S6 (p-S6) was checked by immunofluorescence in tumor tissues. Our results showed that the p-S6 expression was weaker in the ZA group, indicating that ZA could decrease the p-S6 expression in the TSC2-null cell tumor (Fig. 1d). Advanced activation of mTOR pathway makes TSC2-null cells have impaired autophagic flux. We wondered whether ZA could induce autophagy in TSC2-null cells. Firstly, we checked the p-S6 expression in vitro to further confirm ZA affect the activity of mTOR pathway. Our results showed that the p-S6 was partly reduced in the high-concentration ZA group (Additional file 2: Fig. S1A). Moreover, the accumulation of LC3-II was observed in the ZA treatment group (Additional file 2: Fig. S1B). This phenomenon was also confirmed by the immunofluorescence staining of LC3 and LAMP1 (Additional file 2: Fig. S1C). In addition, the GGPP treatment rescued the excessive autophagy in ZA-treated TSC2-null cells (Additional file 2: Fig. S1D). Together, these findings suggest that ZA could cause TSC2-null cell autophagy by inhibiting GGPP.

In order to simulate the clinical features of LAM patients, the mouse model of LAM was re-built with TSC2-null cells. The lung morphology observation showed that TSC2-null cell lesions in mice tended to accumulate around the veins and arteries [there were no other tumor lesions in other organs (Additional file 3: Fig.S2B)]. The mTORC1 activity marker p-S6 was also checked, and the H&E staining was performed. Our results showed significantly increased space in the alveolar airspace (Additional file 3: Fig. S2C) and higher p-S6 expression level (Additional file 3: Fig. S2A) compared with the normal lung structure. Next, tumor growth and the alveolar destruction in LAM mouse model were assessed. We observed that inhibition of tumor growth depended on drug concentrations of ZA (Fig. 1e).

### ZA induces apoptosis of TSC2-null cell via inhibiting GGPP

**Fig. 2:**
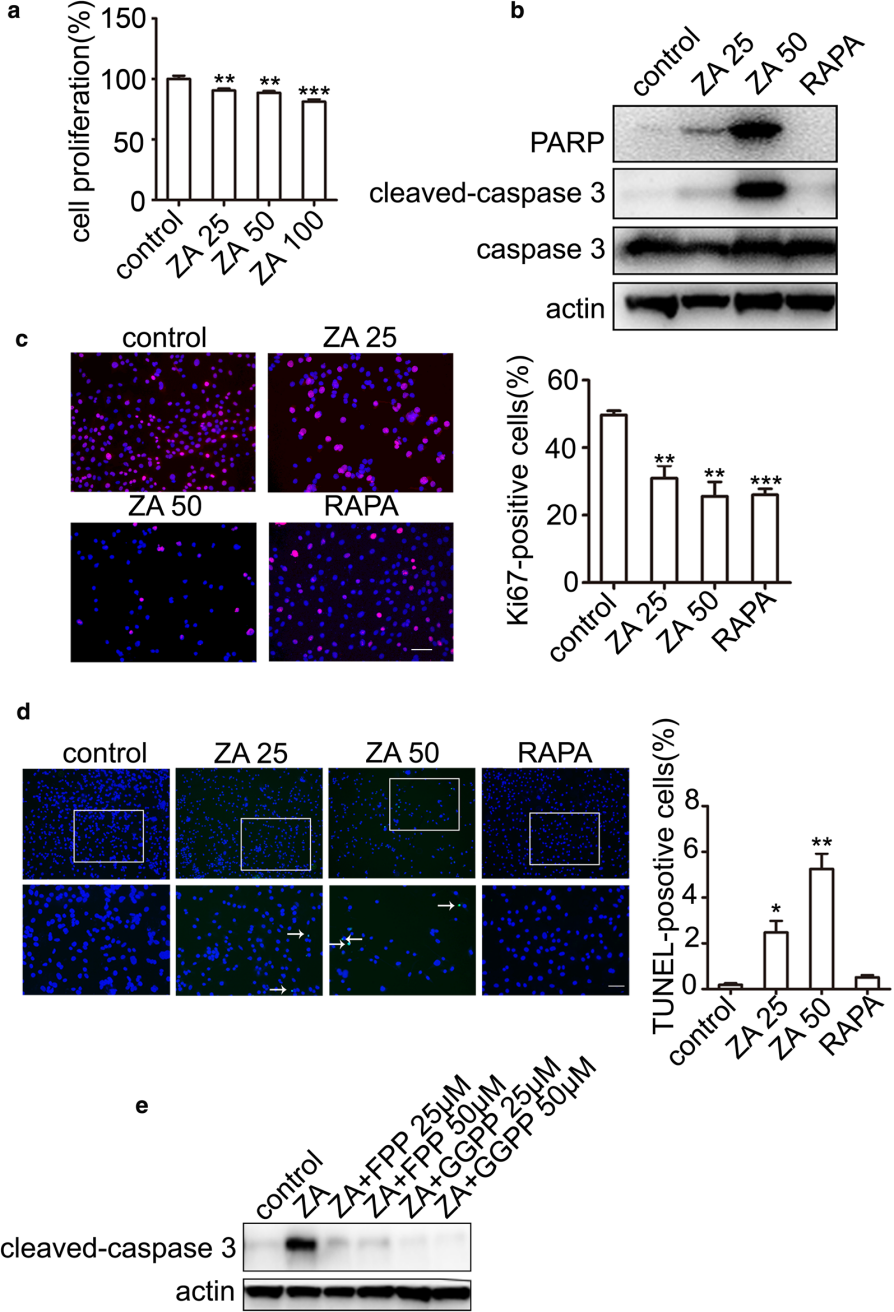
ZA induces TSC2-null cell apoptosis by GGPP. **a** MTT assay of TSC2-null cells treated with control, as well as 25, 50, and 100 μM ZA. **b** Western blot analysis after treatment with ZA (25 and 50 μM, respectively; and 20 nM RAPA for 24 h) in TSC2-null cells. **c** Immunostaining of Ki67 after treatment with ZA (25 and 50 μM; and 20 nM RAPA for 24 h). Immunofluorescence staining was performed and analyzed in independent 3 experiments. **d** Apoptosis of TSC2-null cells treated with ZA assayed using the DeadEnd™ Fluorometric TUNEL System. The green dots represented apoptotic cells, and DAPI (blue) indicated cell nuclei. Scar bar, 100 and 200 μm, respectively. **e** Immunoblotting analysis of apoptosis marker (cleaved-caspase3) after treatment with ZA alone, the combination of ZA and FPP, and the combination of ZA and GGPP, respectively, for 24 h. The experiment was performed for three times. All data were presented as mean ± SEM. Compared with the control group, ** *P* < 0.01, *** *P *< 0.001

To further determinate the antitumor effects of ZA in vitro, TSC2-null cells were treated with indicated concentrations of ZA. We also found that ZA inhibited the TSC2-null cell proliferation, in a dose-dependent manner (Fig. 2a). Moreover, comparing with rapamycin, ZA inhibited cell proliferation and induced apoptosis more effectively (Fig. 2b). These results were also confirmed by the immunofluorescence staining of Ki67 (Fig. 2c) and the DeadEnd™ Fluorometric TUNEL System analysis (Fig. 2d). To assess which subtract of ZA affects the proliferation of TSC2-null cells, FPP and GGPP were added into the culture medium, respectively. Our results showed that the apoptosis could be rescued by the FPP and GGPP both (Fig. 2e). However, FPP could produce GGPP by geranylgeranyl pyrophosphate synthase (GGPPS) in the cells. Treatment of GGPP alone (without FPP) could rescue the inhibitory effect of ZA, suggesting that ZA exerted anticancer effects because of inhibition of GGPP.

### ZA induces apoptosis of TSC2-null cells by inactivating RhoA/YAP pathway

**Fig. 3:**
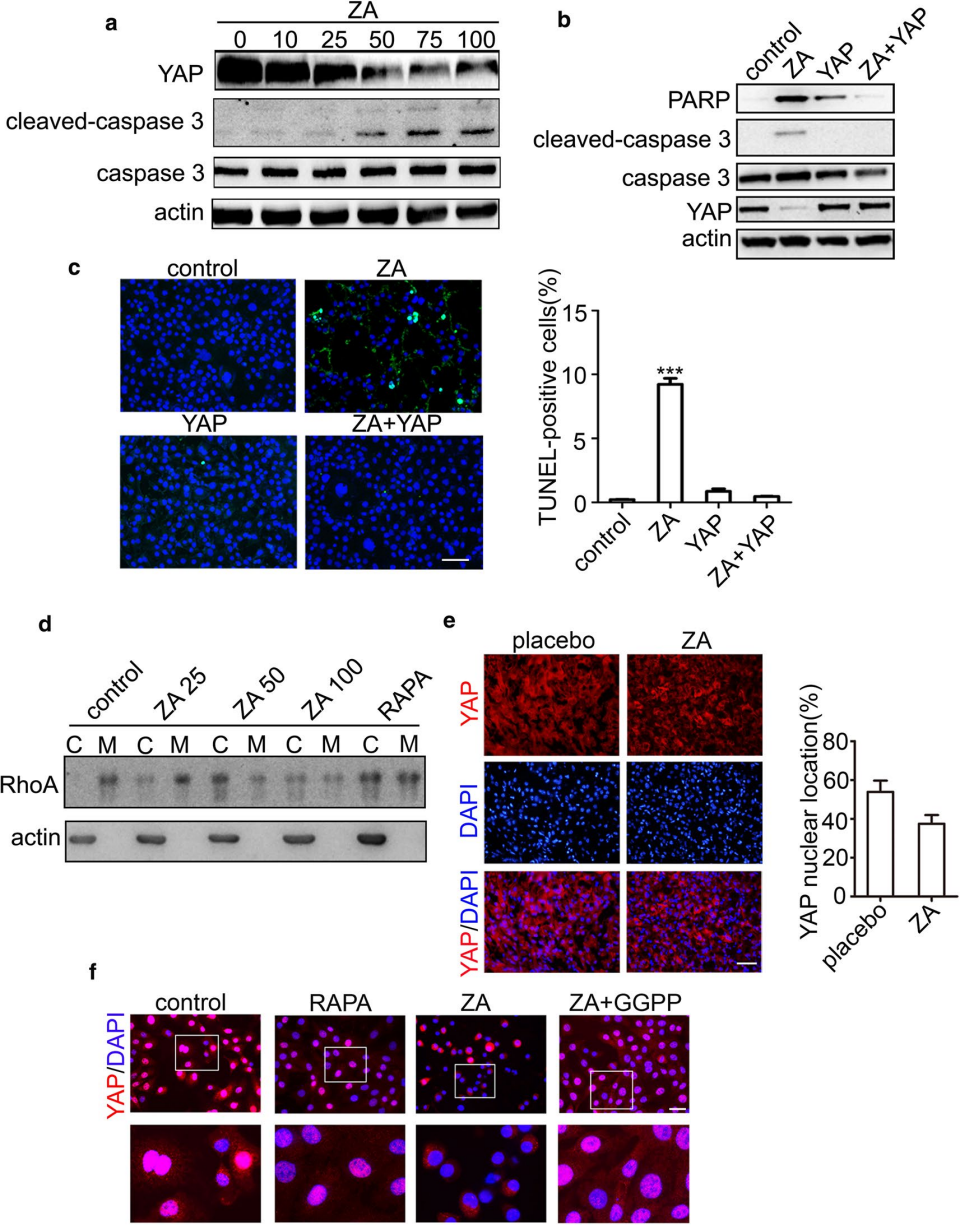
ZA induced apoptosis by inhibiting YAP. **a** Yap, caspase-3, and cleaved caspase-3 levels were determined by the Western blot analysis, after the treatment of 0, 10, 25, 50, 75, and 100 μM ZA, respectively. **b** PARP and cleaved-caspase-3 levels were detected by the Western blot analysis after the treatment with ZA, YAP over-expression plasmid, and ZA and YAP over-expression plasmid, respectively. **c** Apoptosis was determined by TUNEL assay. The green dots represented apoptotic cells, and DAPI (blue) indicated cell nuclei. Scale bar, 100 μm. **d** Immunoblotting detection of RhoA localization at the plasma membrane of TSC2-null cells treated with control, ZA (25, 50, and 100 μM), and rapamycin (20 nM) for 24 h with serum. Scale bar, 50 μm. **e** Immunofluorescence analysis of Yap localization of TSC2-null cells in subcutaneous mouse tumors. Percentages of Yap-nuclear localized cells were calculated in different tumor lesions. Scale bar, 200 μm. **f** Yap translocation was determined by immunofluorescence after treated with rapamycin, ZA, and combination of ZA and GGPP. Scale bar, 50 μm. The experiment was performed for three times. All data were presented as mean ± SEM. Compared with the control group, *** *P *< 0.001

Some study showed that Yap is essential for TSC2-null cell. To specifically validate Yap function could be affected by ZA, the expression levels of Yap was checked after treating with different concentrations of ZA. Our results showed that the Yap expression level was steadily decreased in a dose-dependent manner with ZA treatment (Fig. 3a). Next, we over-expressed YAP in the TSC2-null cells and showed that cleaved-caspase-3 and PARP was sharply attenuated after YAP over-expression (Fig. 3b), indicating that the apoptosis of TSC2-null cells induced by ZA was blunted by the YAP over-expression. These results were further confirmed by detection of TUNEL^+^ apoptotic cells (Fig. 3c). RhoA plays an important role in Yap function. We next checked RhoA expression in the cell membrane and cytoplasm by immunoprecipitation and we found that the expression of RhoA in the cell membrane was reduced (Fig. 3d). Yap expression was also examined in the subcutaneous tumor model by immunofluorescence and showed that the expression of Yap was sharply attenuated in the ZA group (Fig. 3e). Moreover, GGPP can rescue the Yap nuclear translocation in vitro (Fig. 3f). Collectively, these date demonstrate that ZA induces apoptosis of TSC2-null cells by blocking GGPP leading to inhibition of Yap.

### ZA induces autophagy leading to Yap protein degradation in TSC2-null cells

Autophagy usually causes proteins degradation. To check whether the decreased Yap protein expression was caused by autophagy, the mRNA expression of Yap was detected. Our result showed that there was no difference in the Yap mRNA level after the ZA treatment (Additional file 4: Fig. S3C). These results suggest that the Yap protein down-regulation may be due to protein degradation, which may be caused by autophagy, but not decrease of YAP mRNA. Moreover, the ZA treatment could induce an excessive level of autophagy (Additional file 2: Fig. S1D). Furthermore, when the TSC2-null cells were treated with the autophagy inhibitor CQ, the Yap expression and cell proliferation would be partly rescued (Additional file 4: Fig. S3A, B). These results suggest that autophagy induced by ZA in the TSC2-null cells mainly showed the dark-side effects. Based on these findings, we hypothesize that ZA might induce excessive autophagy in the TSC2-null cells, leading to the Yap degradation and eventually causing the cellular apoptosis.

### Combination of ZA and rapamycin inhibits tumor recurrence after drug withdrawal

**Fig. 4:**
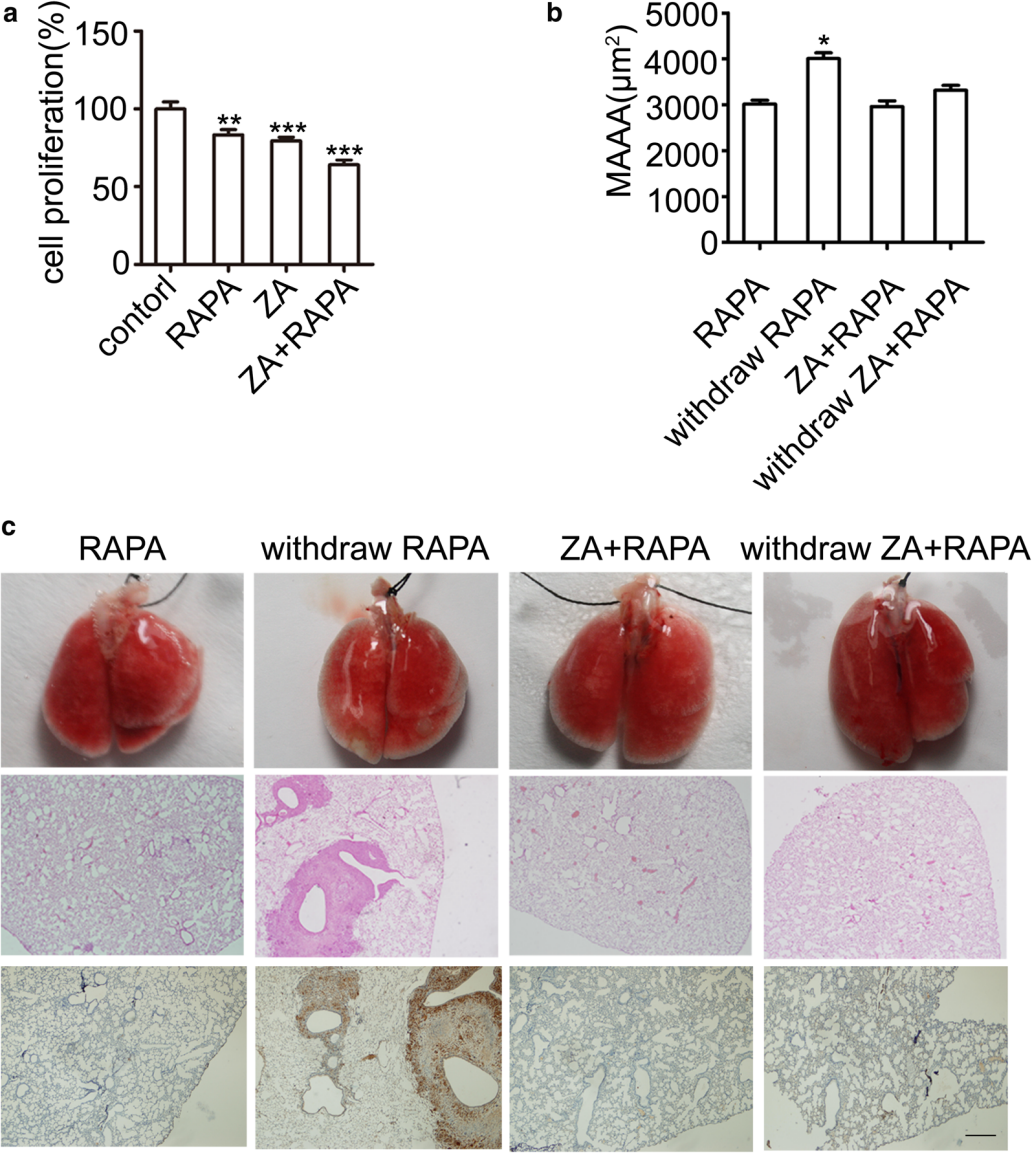
Combination of ZA and rapamycin inhibits tumor recurrence after drug withdrawal. **a** Cell viability was determined by MTT after treated with rapamycin, ZA, and combination of ZA and rapamycin, respectively, for 24 h. **b** Lung lesion was determined in the MAAA in LAM mouse model. **c** H&E analysis of lung lesions and p-S6 staining of lung sections after injection of TSC2-null cells involving rapamycin (RAPA), the combination of RAPA and ZA-injected, and withdrawal therapy, after 30 day (n = 6). Scar bar, 500 μm. The experiment was performed for three times. All data were presented as mean ± SEM. Compared with the control group, *P < 0.05, ** *P* < 0.01, *** *P *< 0.001

Resistance of rapamycin has been described in some tumor cells and clinical cases. In this study, whether the combination of ZA and rapamycin inhibited the tumor growth in LAM mouse model was assessed. The MTT analysis showed that combination of ZA and rapamycin inhibited the growth of TSC2-null cells more effectively (Fig. 4a). Importantly, the combination of ZA and rapamycin showed significant synergistic effects compared with the rapamycin treatment alone, which stopped the tumor recurrence after drug withdrawal, with minor lung lesions (Fig. 4b, c). These results indicate that the combination of ZA and rapamycin could strongly inhibit the tumor progress and prevent the tumor recurrence in the LAM mouse models.

## Discussion

Rapamycin is the only drug approved by FDA in the US and Japan for the clinical treatment of LAM. Lacking effective single therapeutic drugs for LAM makes the combination drug regimens necessary, such as the rapamycin in combination with statins and autophagy inhibitors. Currently, sirolimus (rapamycin) and hydroxychloroquine (autophagy inhibitor) have reached the clinical phase I [13]. The mevalonate pathway metabolites are essential for cancer cell survival and growth. ZA and simvastatin are inhibitors of the mevalonate pathway, with different target sites. The combination of rapamycin and simvastatin exhibits efficacy in LAM mouse models [25]. However, tumor recurrence after rapamycin treatment is a common problem in the clinical treatment of LAM, and there has been no good solution. In this study, for the first time, we validated that the combination of ZA and rapamycin, targeting on the mTOR and mevalonate pathways, could eliminate the tumors and inhibit tumor recurrence in the LAM mouse models.

Among small GTPases, RhoA and Rheb are essential parts of mTORC1 and mTORC2, respectively [27]. In addition, Ras activates the PI3K/AKT pathway, associated with the mTOR pathway [28]. ZA is an inhibitor of the mevalonate pathway, which inhibits the prenylation of small GTPases (i.e., the Ras, Rho, and Rac) [29]. We inferred that ZA could be an effective drug for apoptosis of TSC2-null cells. Indeed, our results showed that ZA not only inhibited the proliferation (just like rapamycin), but also promoted the apoptosis, of TSC2-null cells. More importantly, the combination of ZA and rapamycin absolutely eliminated the tumor, and no tumor recurrence occurred after drug withdrawal in the LAM mouse models. All these data suggests that the combination treatment of bisphosphonate and rapamycin provides new treatment method for LAM.

How *TSC2* mutation leads to abnormal proliferation of LAM cells has not yet been fully elucidated. Some researches demonstrated that the excessive proliferation of TSC2-null cells is due to the accumulation of Yap in the nucleus [30, 31]. The nuclear translocation of Yap is regulated by the RhoA protein, which requires the modification of GGPP [32]. We speculate that ZA might inhibit the Yap activity by blocking GGPP. The expression of Yap after ZA treatment was examined. Our results showed that, as the increase of ZA concentrations, the expression levels of Yap were gradually reduced in the TSC2-null cells. Moreover, the YAP over-expression could reverse the apoptosis induced by ZA. These results demonstrate that ZA could induce apoptosis of TSC2-null cells by inhibiting Yap. Our results also showed that the RhoA protein bound on the cell membrane was significantly decreased, along with the increase concentrations of ZA. Moreover, the autophagic process was also triggered by the ZA treatment.

The limitation of our study is that the effects of ZA in patient LAM cells have not been verified. In recent years, high medical research and development costs limit the development of many orphan drugs, but the development of new clinical drugs can shorten the development cycle and save the development costs. ZA has been used in the clinical treatment of osteoporosis, with validated safety [33].

## Conclusion

Our results showed that ZA affected the nuclear degradation of Yap and caused apoptosis through the protein prenylation. Our research provides new multidrug combination treatment of bisphosphonate and mTOR inhibitors for the mevalonate and mTOR pathways. These findings suggest that ZA might represent a promising pharmaceutical therapeutic drug for LAM.

## Supplementary information

**Additional file 1: Table S1.** Antibodies and chemicals.

**Additional file 2: Fig. S1.** ZA induces autophagy in TSC2-null cells. (A) Phosphorylated S6 and Yap expression detected by immunoblotting analysis in TSC2-null cells treated with ZA (25, 50, and 100 μM, respectively) for 24 h. (B) LC3 expression was detected by immunoblotting analysis in TSC2-null cells treated with ZA (25, 50, 75, and 100 μM, respectively) for 24 h. (C) Localization of LC3 and LAMP1 was analyzed by immunofluorescence in TSC2-null cells treated with ZA (25 and 50 μM, respectively) alone and RAPA (20 nM) for 24 h. (D) Localization of LC3 and LAMP1 was analyzed by immunofluorescence in TSC2-null cells treated with ZA (50 μM), RAPA (20 nM), and combination of ZA (50 μM) and GGPP (20 μM) for 24 h. The experiment was performed for three times. All data were presented as mean ± SEM. Compared with the placebo, * *P* < 0.05.

**Additional file 3: Fig. S2.** LAM mouse model establishment. (A) Analysis of lung lesions and immunohistochemical staining of p-S6 in LAM mouse models after tail vein injection of TSC2-null cells for 30 day. Scar bar, 50, 200, and 500 μm, respectively. (B) H&E staining of liver, heart, and kidney after tail vein injection of TSC2-null cells for 30 day. Scar bar, 500 μm. (C) Analysis of MAAA of lungs after tail vein injection of TSC2-cells for 30 day.

**Additional file 4: Fig. S3.** ZA induce autophagy leading to Yap protein degradation in TSC2-null cells. (A) Cell viability was determined by MTT after treated with rapamycin, ZA, and combination of ZA and CQ, respectively, for 24 h. (B) Yap translocation was determined by immunofluorescence after treated with rapamycin, ZA, and combination of ZA and CQ, for 24 h. Scale bar, 50um. (C) Quantitative real-time PCR analysis of YAP mRNA expression after ZA treatment with 25uM, 50uM, 100uM, for 24 h. The experiment was performed for three times. All data were presented as mean ± SEM. Compared with the placebo, * P < 0.05.

## Data Availability

Not applicable.

## Abbreviations

ZA: Zoledronic acid
LAM: Lymphangioleiomyomatosis
TSC: Tuberous sclerosis complex
