# Supplementary material for: Zoledronic acid inhibits TSC2-null cell tumor growth via RhoA/YAP signaling pathway in mouse models of lymphangioleiomyomatosis
Source: Cancer Cell Int. 2020 Feb 10;20:46. doi: 10.1186/s12935-020-1131-4 (PMC7011352; doi:10.1186/s12935-020-1131-4)
Supplement: Supplementary file 1 — Additional file 1: Table S1. Antibodies and chemicals. [file 12935_2020_1131_MOESM1_ESM.docx]

**Additional file 4: Table S1** Antibodies and chemicals.

| **Chemicals** | **Origin** | **Application** | **Dilution** | **Reference** |
| --- | --- | --- | --- | --- |
| Zoledronic acid (ZA) | Selleck chemicals |  |  | S1314 |
| Rapamycin(sirolimus) | Selleck chemicals |  |  | S1039 |
| Geranylgeranylpyrophosphate ammonium salt(GGPP) | Sigma Aldrich |  |  | G6025 |
| Farnesyl pyrophosphate ammonium salt(FPP) | Sigma Aldrich |  |  | F6892 |
| **Antibodies** | **Origin** | **Application** | **Dilution** | **Reference** |
| P-S6 (ser235/236) | Cell signaling technology | WB | 1:1000 | 4858 |
| YAP | Cell signaling technology | WB/IF | 1:1000  1:200 | 14074 |
| RhoA | Santa Cruz | WB/IP | 1:500 | sc-418 |
| Caspase 3 | Cell signaling technology | WB | 1:1000 | 9662 |
| LAMP1 | Cell signaling technology | IF | 1:200 | [15665](https://www.cst-c.com.cn/products/primary-antibodies/lamp1-d4o1s-mouse-mab/15665?site-search-type=Products&N=4294956287&Ntt=lamp1&fromPage=plp) |
| LC3 | Cell signaling technology | WB/IF | 1:1000  1:200 | 4108 |
| Ki67 | Cell signaling technology | IF | 1:200 | 9129 |
| PARP | Cell signaling technology | WB | 1:1000 | [9532](https://www.cst-c.com.cn/products/primary-antibodies/parp-46d11-rabbit-mab/9532?site-search-type=Products&N=4294956287&Ntt=parp&fromPage=plp) |
| β-actin | Proteintech | WB | 1:1000 | 66009-1-Ig |
| α-tubulin | Proteintech | WB | 1:1000 | 66031-1-Ig |
| Alex Fluor 594 | [Thermo Fisher Scientific](https://www.thermofisher.com/cn/zh/home/brands/molecular-probes/key-molecular-probes-products.html) | IF | 1:500 | A-11032 |
| Alex Fluor 488 | [Thermo Fisher Scientific](https://www.thermofisher.com/cn/zh/home/brands/molecular-probes/key-molecular-probes-products.html) | IF | 1:500 | A-11034 |
| DAPI | [Thermo Fisher Scientific](https://www.thermofisher.com/cn/zh/home/brands/molecular-probes/key-molecular-probes-products.html) | IF | 3 μM | D1306 |
| **Kits** | **Origin** | **Application** | **Dilution** | **Reference** |
| MTT | KeyGENBioTECH | Cell viability |  | KGA311 |
| Dead End^TM^ Fluorometric TUNEL System | Promega | IF |  | G3250 |
