# Supplementary figures and images for: Zoledronic acid inhibits TSC2-null cell tumor growth via RhoA/YAP signaling pathway in mouse models of lymphangioleiomyomatosis
Source: Cancer Cell Int. 2020 Feb 10;20:46. doi: 10.1186/s12935-020-1131-4 (PMC7011352; doi:10.1186/s12935-020-1131-4)

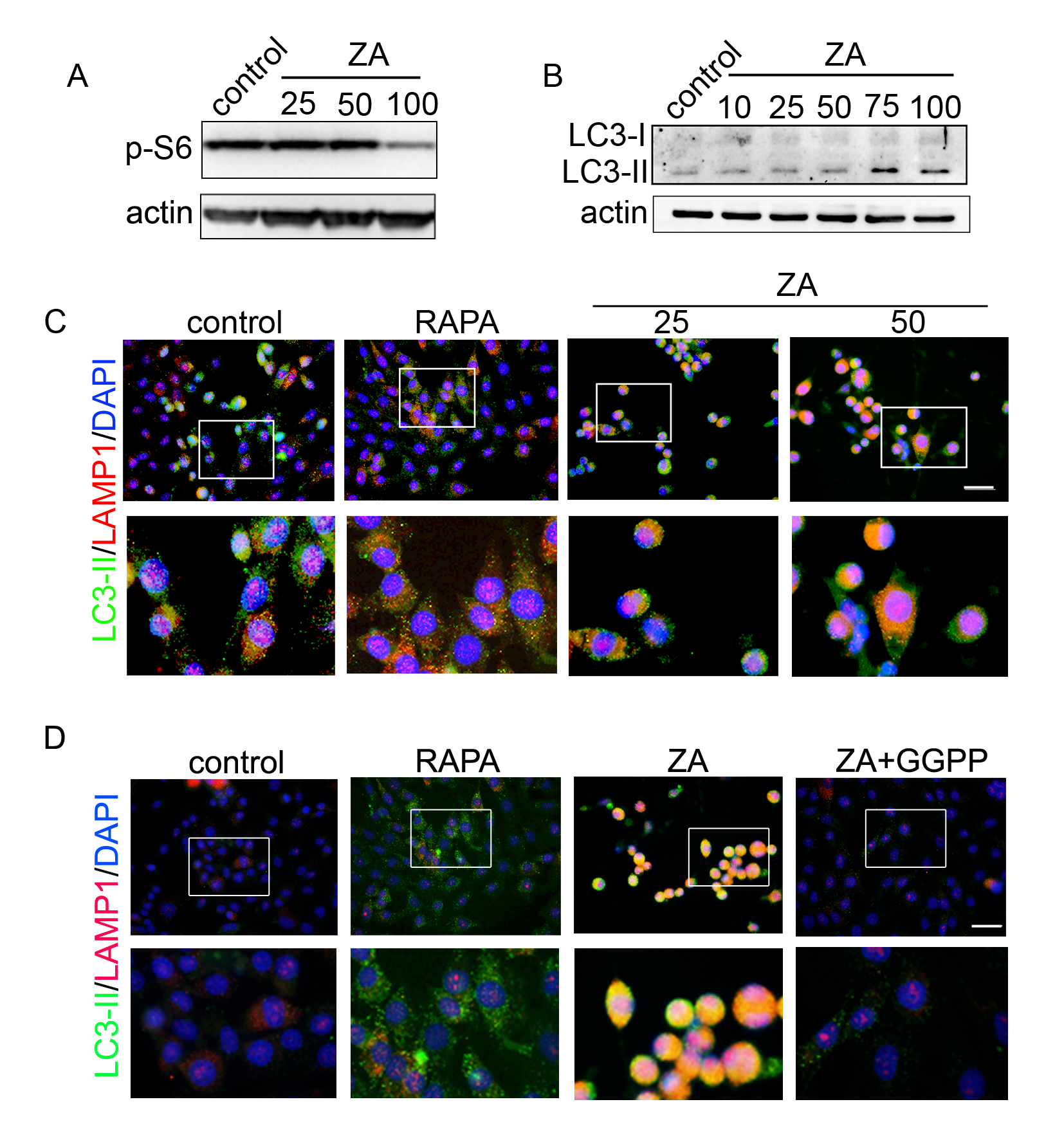

Supplement: Supplementary file 2 — Additional file 2: Fig. S1. ZA induces autophagy in TSC2-null cells. (A) Phosphorylated S6 and Yap expression detected by immunoblotting analysis in TSC2-null cells treated with ZA (25, 50, and 100 μM, respectively) for 24 h. (B) LC3 expression was detected by immunoblotting analysis in TSC2-null cells treated with ZA (25, 50, 75, and 100 μM, respectively) for 24 h. (C) Localization of LC3 and LAMP1 was analyzed by immunofluorescence in TSC2-null cells treated with ZA (25 and 50 μM, respectively) alone and RAPA (20 nM) for 24 h. (D) Localization of LC3 and LAMP1 was analyzed by immunofluorescence in TSC2-null cells treated with ZA (50 μM), RAPA (20 nM), and combination of ZA (50 μM) and GGPP (20 μM) for 24 h. The experiment was performed for three times. All data were presented as mean ± SEM. Compared with the placebo, * P < 0.05. [file 12935_2020_1131_MOESM2_ESM.tif]

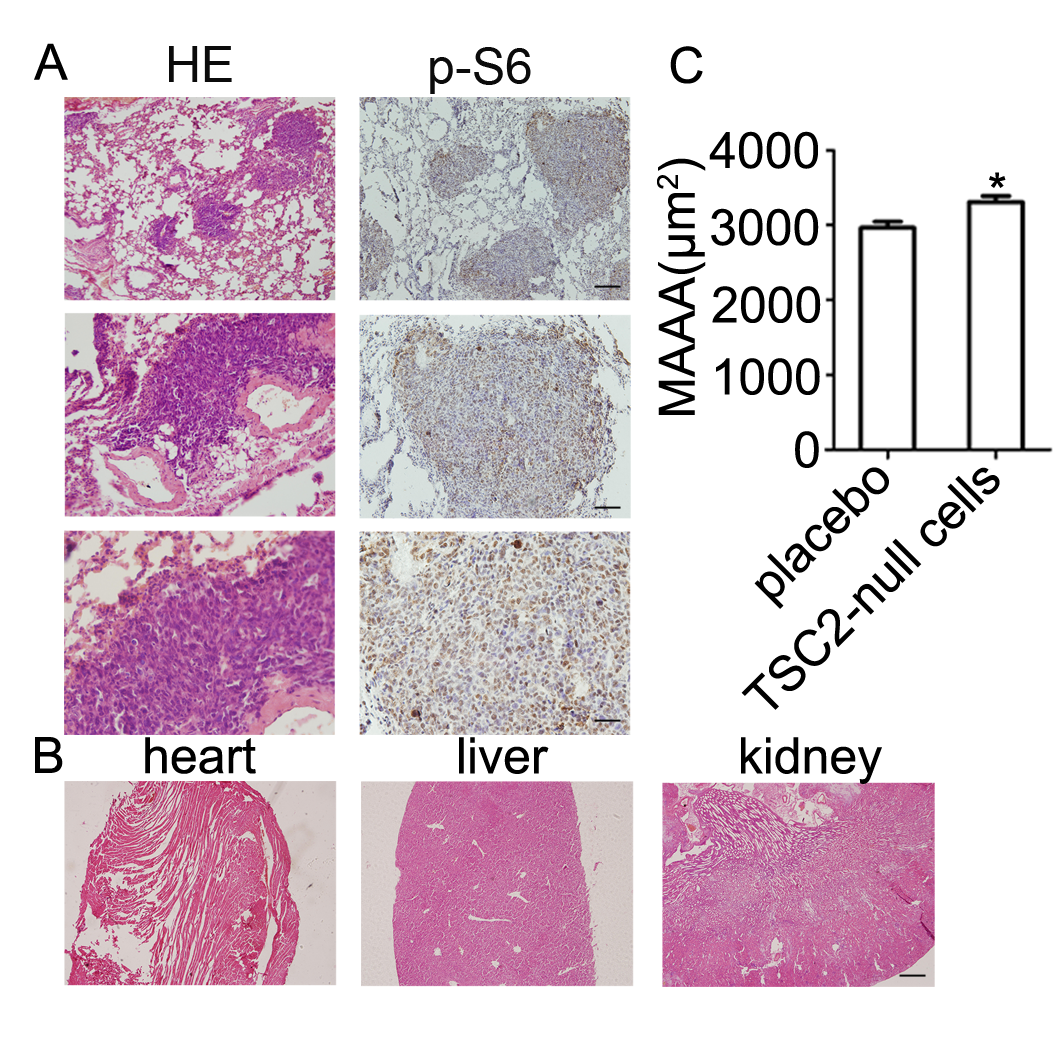

Supplement: Supplementary file 3 — Additional file 3: Fig. S2. LAM mouse model establishment. (A) Analysis of lung lesions and immunohistochemical staining of p-S6 in LAM mouse models after tail vein injection of TSC2-null cells for 30 day. Scar bar, 50, 200, and 500 μm, respectively. (B) H&E staining of liver, heart, and kidney after tail vein injection of TSC2-null cells for 30 day. Scar bar, 500 μm. (C) Analysis of MAAA of lungs after tail vein injection of TSC2-cells for 30 day. [file 12935_2020_1131_MOESM3_ESM.tif]

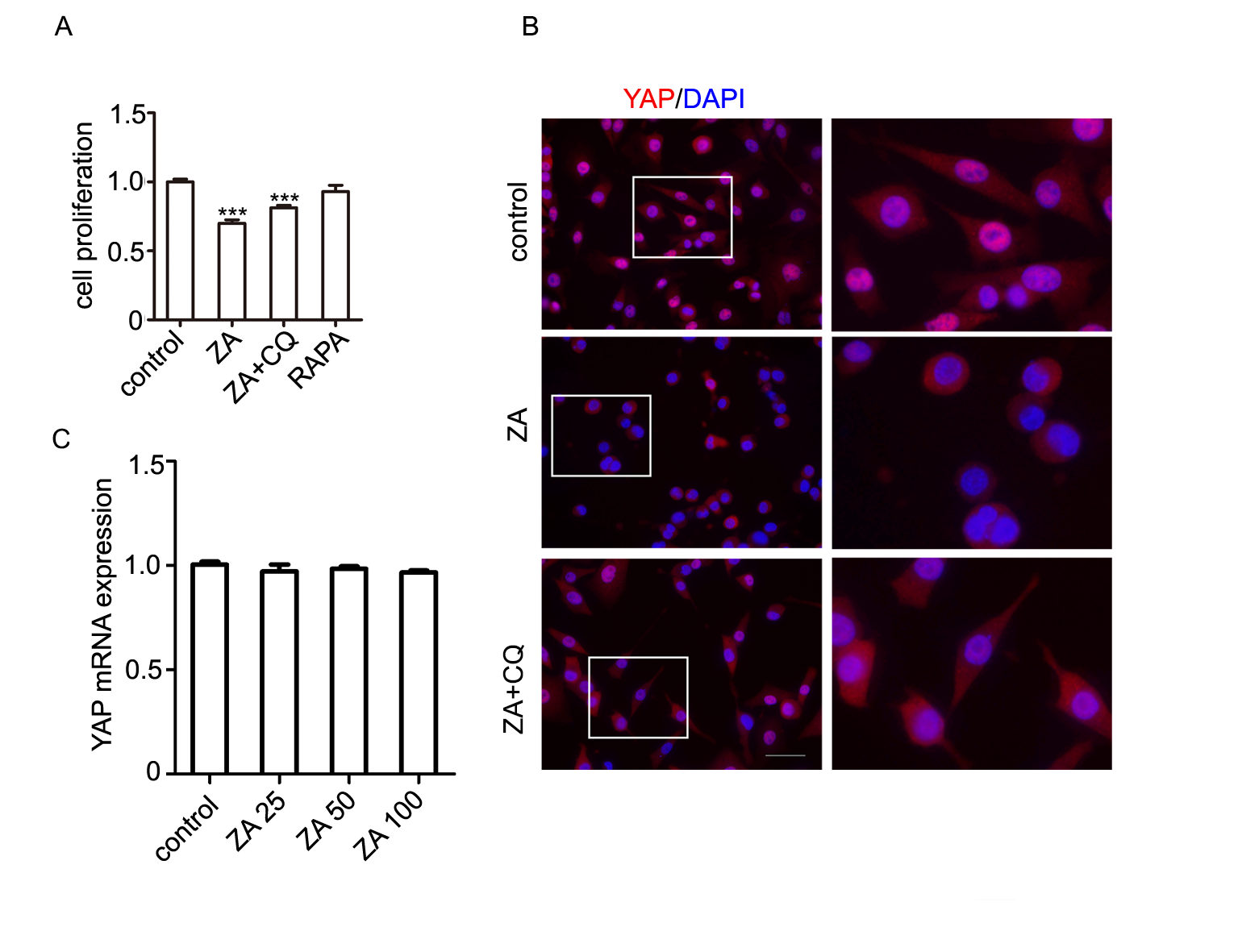

Supplement: Supplementary file 4 — Additional file 4: Fig. S3. ZA induce autophagy leading to Yap protein degradation in TSC2-null cells. (A) Cell viability was determined by MTT after treated with rapamycin, ZA, and combination of ZA and CQ, respectively, for 24 h. (B) Yap translocation was determined by immunofluorescence after treated with rapamycin, ZA, and combination of ZA and CQ, for 24 h. Scale bar, 50um. (C) Quantitative real-time PCR analysis of YAP mRNA expression after ZA treatment with 25uM, 50uM, 100uM, for 24 h. The experiment was performed for three times. All data were presented as mean ± SEM. Compared with the placebo, * P < 0.05. [file 12935_2020_1131_MOESM4_ESM.tif]
